# Naturally Occurring Canine Invasive Urinary Bladder Cancer: A Complementary Animal Model to Improve the Success Rate in Human Clinical Trials of New Cancer Drugs

**DOI:** 10.1155/2017/6589529

**Published:** 2017-04-09

**Authors:** Christopher M. Fulkerson, Deepika Dhawan, Timothy L. Ratliff, Noah M. Hahn, Deborah W. Knapp

**Affiliations:** ^1^Department of Veterinary Clinical Sciences, Purdue University, West Lafayette, IN 47907, USA; ^2^Department of Comparative Pathobiology, Purdue University, West Lafayette, IN 47907, USA; ^3^Purdue University Center for Cancer Research, Purdue University, West Lafayette, IN 47907, USA; ^4^Department Oncology and Urology, Johns Hopkins University School of Medicine and Sidney Kimmel Comprehensive Cancer Center, Baltimore, MD 21287, USA

## Abstract

Genomic analyses are defining numerous new targets for cancer therapy. Therapies aimed at specific genetic and epigenetic targets in cancer cells as well as expanded development of immunotherapies are placing increased demands on animal models. Traditional experimental models do not possess the collective features (cancer heterogeneity, molecular complexity, invasion, metastasis, and immune cell response) critical to predict success or failure of emerging therapies in humans. There is growing evidence, however, that dogs with specific forms of naturally occurring cancer can serve as highly relevant animal models to complement traditional models. Invasive urinary bladder cancer (invasive urothelial carcinoma (InvUC)) in dogs, for example, closely mimics the cancer in humans in pathology, molecular features, biological behavior including sites and frequency of distant metastasis, and response to chemotherapy. Genomic analyses are defining further intriguing similarities between InvUC in dogs and that in humans. Multiple canine clinical trials have been completed, and others are in progress with the aim of translating important findings into humans to increase the success rate of human trials, as well as helping pet dogs. Examples of successful targeted therapy studies and the challenges to be met to fully utilize naturally occurring dog models of cancer will be reviewed.

## 1. Introduction

With more than 7.5 million deaths from cancer worldwide each year, there is an ever increasing need to develop better cancer therapies, along with strategies to make cancer care more accessible. Even with tremendous advances in pharmaceutical science and technology, nine out of ten human clinical trials of new cancer therapies fail [1]. One of the major reasons for these failures is the inability of preclinical models to predict the safety and efficacy of new cancer drugs in humans [1]. In in vitro systems, carcinogen-induced, engraftment, and genetically engineered mouse models are instrumental and essential in cancer research [2–7], but they do not possess the collective features (cancer heterogeneity, molecular complexity, invasion, metastasis, and immune cell response) critical to predict success or failure of emerging therapies in humans [1].

The need for relevant cancer models has never been greater. With advances in sequencing methods and genomic analyses, the number of therapeutic candidates with sound biologic rationale justifying investigation in humans continues to grow [8–16]. However, with the rapidly expanding number of combinations of targets and mechanisms of action, the cancer research community has entered an uncharted era in which the number of cancer patients (especially those with uncommon cancer types) is insufficient for testing even part of the new therapeutic approaches. Hence, there is an essential need to develop and optimize preclinical animal models to rapidly facilitate more accurate predictors of therapeutic success in approaches ultimately chosen to take forward into humans. With the resurgence of immunotherapies and the understanding of the immune system's role in many types of therapies [16–18], it is critical that animal models also possess a level of immunocompetence similar to that in human cancer patients. As summarized in this review, and using invasive urinary bladder cancer as an example, there is compelling evidence that pet dogs with specific forms of naturally occurring cancer can provide crucially needed relevant animal models to complement other models in preclinical research to help improve the success rate in human clinical trials [19, 20].

## 2. Challenges in Invasive Urinary Bladder Cancer and a Need for Animal Model Research

There is a dire need for relevant animal models for research to improve the treatment and management of humans with invasive urinary bladder cancer (InvUC). In humans, InvUC is defined as high-grade UC that has invaded to the depth of the lamina propria (T1 tumors) or beyond (T2–T4 tumors; also commonly called muscle invasive bladder cancer) [21]. Even in patients where the local infiltration is limited to the lamina propria (T1 tumors), that is, not yet into the muscle, InvUC represents an aggressive, often fatal cancer [21]. It should be noted that of all human bladder cancer, up to 80% of cases are noninvasive, but the invasive cancers represent the most challenging form of the cancer to be treated. Current challenges faced by patients with InvUC in the United States, for example, include the following: (1) 50% fatality rate and >16,000 deaths per year, (2) reduced quality of life from both the cancer and its treatment (cystectomy, radiotherapy, and chemotherapy), and (3) financial costs ($150,000 median cost of care per patient; $17 billion per year lost due to untimely deaths from InvUC) [21–28]. In addition, of the 550,000 people living with nonmuscle invasive cancer in the US, there is a risk of progression to muscle-invasive stages requiring cystectomy in 10–30% of the patients. The need for better InvUC drugs is crucial as drugs are now being applied in multiple settings including the BCG unresponsive, neoadjuvant and adjuvant perioperative, bladder sparing, and metastatic populations [8, 22–27]. With rare exceptions, relapse after treatment for metastatic disease is inevitable, with few patients achieving durable benefit from the second line treatment [29]. There is, however, hope for vast improvement in the success of InvUC therapy with emerging molecularly targeted drugs, epigenetic-targeted drugs, and immunotherapies, provided that animal models can meet research demands [9, 10, 15–18, 25].

Animal models are considered a key to all bladder cancer research, and several instrumental model systems have been defined. Carcinogen-induced tumors in laboratory animals have been used to understand features of bladder tumor development and drug effects at different stages of the process in immunocompetent hosts [2–5]. In syngeneic and immunodeficient mice, tumor cells can be implanted subcutaneously or orthotopically in the bladder to evaluate drug activity. Different types of cells including tumor cells, epithelial cells, stromal cells, and embryonic urogenital sinus mesenchymal cells can be implanted under the renal capsule to evaluate drugs and to study epithelial-stromal interactions [2, 3, 30]. Genetically engineered mouse models (GEMs) have been widely used in other types of cancer to analyze tumor phenotype, to study some subtypes, to investigate candidate genes and signaling pathways, and to test drugs, and GEMs are beginning to be used more in bladder cancer research [2–4]. GEMs for bladder tumors include those with altered *pRB* and/or *p53* [31, 32]; *Pten* with or without *p53* [33, 34]; *Kras* and *Hras* [35, 36]; *Egfr* [37]; *p21* [35]; *IL17*, *IL12*, *IL23*, and *IFNγ* [38–40]; and others [2–4]. Patient-derived xenograft models are also promising tools to study drug effects on an individual's tumor, and these are now being described for bladder cancer [41, 42]. As with other cancers, however, these animal models of InvUC do not meet the increasing demands of the complex cancer therapies being developed to treat the aggressive heterogenous cancer in humans [8–18, 43–52]. But there is growing evidence that an intriguing complementary animal model, naturally occurring InvUC in dogs, could help address these demands [19, 53, 54].

## 3. Naturally Occurring Canine Invasive Bladder Cancer and Relevance to Human Invasive Bladder Cancer

The vast majority of naturally occurring bladder cancer in dogs consists of InvUC [19]. It should be noted that the nomenclature for the T stages for canine bladder cancer differs somewhat from that used in humans [19]. In dogs, “T1 tumors” are superficial and have not invaded the lamina propria as T1 tumors in humans have. Therefore, in dogs, invasive cancer is categorized as T2 or T3. There is another difference in the staging system between dogs and humans. In dogs, T2 tumors are any tumors that have invaded the muscularis, while T3 tumors are those that have invaded neighboring organs; thus, T2 and T3 tumors comprise the invasive UC reported here. In humans, T2 tumors have invaded the muscle, T3 tumors have invaded the deep fat under the muscle, and T4 tumors have invaded the neighboring organs. Nonmuscle invasive bladder cancer, which is very common in people with bladder cancer, is rare in dogs [19]. Canine bladder cancer offers a model of the more lethal bladder cancer biology in humans, InvUC. Canine InvUC mimics human InvUC in the following: (1) pathology including cellular features, tumor heterogeneity, and infiltrating immune cells [19, 20, 55–62]; (2) local invasion and patterns of distant metastases (lungs and other organs) in >50% of individuals [19]; and (3) response to cisplatin [63–65], carboplatin [66], vinblastine [67, 68], doxorubicin [69], and gemcitabine [70] (summarized in Table 1). Further similarities between canine and human InvUC include shared druggable mutations and pathway variants, epigenetic targets, and evidence for gene patterns of molecular subtypes (basal and luminal) [71–78]. In microarray analyses of genes differentially expressed between canine bladder with InvUC and normal canine bladder, two distinct clusters were identified in the InvUC samples [71]. A recent reanalysis of the data comparing findings to a list of >600 genes that segregate human InvUC into luminal and basal subtypes demonstrated that these two clusters in canine InvUC align closely with luminal and basal expression patterns in humans. Pertinent to in vitro studies, the establishment of canine InvUC cell lines has been reported [79, 80].

Although the molecular characterization of canine InvUC is in the early stages, intriguing similarities to human InvUC have been noted. Mutations in several genes implicated in human InvUC (*EGFR* [9, 50, 81], *CDKN2B* [82, 83], *PIK3CA* [9, 82], *BRCA2* [84], and *NFkB* [85]) have been identified in dogs [72]. *EGFR* overexpression, a particularly interesting target, has been confirmed by immunohistochemistry (IHC) in 73% of canine InvUC cases, similar to that in humans, and a canine trial of an EGFR-targeted compound is ongoing [71, 81]. The expression of estrogen and androgen receptors has also been reported in canine InvUC [19]. In addition, in microarray studies (canine normal bladder, canine InvUC, and human microarray data—GEO database), there were >400 genes differentially expressed between normal and tumor in both dogs and humans [71, 73].

Although there are marked similarities between canine and human InvUC, expected differences do occur (Table 1). The male : female ratio is ~2 : 1 in humans with InvUC [21] but ~0.5 : 1 in dogs affected by InvUC [19], although most dogs studied were spayed or neutered. In an intriguing molecular difference between canine and human InvUC, the majority of canine InvUCs carry a mutation in the MAPK signaling pathway (dog homologue of *BRAF*^V600E^) reported in several human cancers [72, 86, 87]. While *BRAF* mutations are rare in human InvUC, other activating mutations in the MAPK pathway occur in ~30% of cases [9]. The finding of the *BRAF* mutation in canine InvUC opens the door for research in dogs with bladder cancer to be applied across many types of human cancer.

Canine clinical trials of new cancer drugs in which dogs continue life as pets while participating in a trial are well accepted and are considered a win-win scenario that benefits each participating dog and generates knowledge to help people and pet dogs [19, 88, 89]. Although InvUC only comprises 2% of all dog cancers, approximately six million new canine cancer cases are diagnosed yearly in the US, resulting in an ample number of dogs with InvUC available for translational research [90]. A compelling question of any model is the extent to which drug effects in the model will predict drug effects in humans. While this proof-of-concept work has been limited to date, our group has published an unexpected beneficial drug effect first identified in dogs with InvUC that was then found in an exploratory human trial. Briefly, cyclooxygenase (COX) inhibitors have had intriguing antitumor effects and chemotherapy-enhancing effects on dogs with InvUC [19, 63, 65, 68, 91–93]. Given as single agents, COX inhibitors induce remission in 18–20% of dogs with InvUC and cause tumor stabilization in approximately 50–55% of dogs [19, 91]. The addition of COX inhibitors substantially enhances the remission rate of cisplatin (20% with the single agent cisplatin, 50–70% with combined drugs) [63, 65, 92] and vinblastine (23% with the single agent vinblastine, 58% with combined drugs) [68]. In a neoadjuvant trial of a COX-2 inhibitor, celecoxib, given between cystoscopic diagnosis and cystectomy in humans, the same biological effects associated with COX inhibitor-induced remission in dogs were found in humans receiving celecoxib [94].

Another aspect of InvUC in dogs which offers key opportunities for translational research, as well as management of the cancer in dogs, is the very strong dog breed-associated risk. Scottish Terriers have an 18–20-fold increased risk for developing InvUC compared to mixed breed dogs, and Eskimo Dogs, Shetland Sheepdogs, West Highland White Terriers, Keeshonds, Samoyeds, and Beagles have a 3–6-fold increased risk [19]. This offers an unparalleled setting to study heritable risk and gene-environment interactions leading to InvUC and to study early intervention strategies [19, 20, 76, 95–97]. In Scottish Terriers, dogs exposed to lawn chemicals had a 7-fold higher risk of bladder cancer than those not exposed [95]. In humans, approximately half of bladder cancer is due to first-hand cigarette smoke [21]. In dogs across all breeds, a link to second-hand smoke has not been proven, but studies in specific high-risk breeds are indicated. Although heritable factors are thought to play an important role in InvUC risk in humans, groups of humans with this level of heritable risk for the cancer have not been identified due to the tremendous genetic diversity in humans. Once heritable factors are identified in dogs, those same factors or related factors and mechanisms could be investigated in humans [98]. Of equal importance, the strong dog breed-associated risk for InvUC is defining groups of dogs in which to study screening protocols, methods for early detection, and early intervention strategies with promising work ongoing [19, 20, 76, 95–98].

## 4. Examples of Targeted Therapy Research Utilizing the Canine Bladder Cancer Model

### 4.1. Folate-Targeted Therapy

There are published examples of studies in dogs with InvUC which are aimed at translating into humans. One example involves folate-targeted therapy, an approach which stems from the finding that there is much higher uptake of folate (vitamin B9) and folate drug conjugates in certain cancers than in normal tissues [99]. Upregulation of folate receptors (FRs), especially FR*α*, has been noted in several forms of human cancer, while the expression of similar FRs in normal tissues is limited [99, 100]. Although folate-targeted therapy has been evaluated in humans with ovarian cancer and other solid tumors [101, 102], this therapeutic approach has not yet been studied in human bladder cancer. To determine the potential value of folate-targeted therapy in human InvUC, research was conducted to determine FR expression in the human cancer and in the canine InvUC model and the safety and activity of folate-targeted therapy in dogs with InvUC. In dogs, FR expression was detected in 78% of primary InvUC and 80% of nodal metastases by IHC (PU17, polyclonal antibody, Endocyte, West Lafayette, IN) [103]. Scintigraphy was used to confirm folate uptake in primary and metastatic lesions of dogs with InvUC. FR expression was also detected in human InvUC, although further work is required to determine the frequency of the expression. The FR expression in human InvUC was similar to that in dogs when using the PU17 antibody for IHC, but immunoreactivity to a human monoclonal antibody (mab343) was less frequent [103].

A dose escalation study of folate-targeted vinblastine (EC0905, Endocyte) was conducted in dogs with biopsy-confirmed, FR-positive InvUC [103]. As in all clinical trials in dogs at our institution, the study was approved by the Purdue Animal Care and Use Committee, and informed pet owner consent in writing was required for all participating dogs. The maximum tolerated dose (MTD) of EC0905 in dogs (0.25 mg/kg IV weekly) was determined with neutropenia occurring at higher doses. With informative laboratory dog work done before the pet dog trial, only 10 pet dogs with InvUC were required to establish the MTD. In 10 dogs, the tumor responses included partial remission (≥50% reduction in tumor volume) in 5 dogs and stable disease (<50% change in tumor volume) in 4 dogs. The tumor response could not be determined in one dog. With these initial promising findings, a more extensive trial of folate-targeted therapy in dogs with InvUC is ongoing. Demonstrating the benefit in dogs could offer the justification to expand the human application of folate-targeted therapy to bladder cancer, as well as the other cancers currently being investigated.

### 4.2. Epigenetic-Based Therapies—Demethylating Agent Trials

Another example of targeted therapy research in canine bladder cancer with a translational goal involves drugs aimed at epigenetic changes that lead to cancer development and progression in the absence of DNA mutations [104–108]. One of the key epigenetic events is the aberrant methylation in the promoter region of tumor suppressor genes, resulting in gene silencing. Aberrant DNA methylation has been identified in multiple genes in human InvUC [107, 108]. DNA methyltransferase 1 (DNMT1), which is a key player in the aberrant methylation process, has been noted to be overexpressed in human and canine InvUC [73, 109]. When considering the design of clinical trials of demethylating agents in humans, key valuable information that could be rapidly obtained from dogs includes efficacy, safety, and treatment scheduling. With the need for this information, a clinical trial of the demethylating agent, 5-azacitidine (5-AzaC), in dogs with InvUC was conducted [74]. Doses were escalated in 2 different dose schedules (daily treatment for 5 sequential days for one cycle per month or daily treatment for 5 sequential days for two cycles per month). Of 18 dogs evaluable for tumor response, partial remission, stable disease, and progressive disease were observed in 4 (22.2%), 9 (50.0%), and 4 (22.2%) dogs, respectively. Although remission is certainly preferred, durable stable disease (i.e., cancer control) of bladder cancer can also be a very beneficial response. The MTD in each treatment schedule was defined, with neutropenia occurring at higher doses. Consistent 5-AzaC urine levels (205–857 ng/ml) were measured.

While 5-AzaC had promising activity in dogs with InvUC, the route of administration (subcutaneous injection) required in-hospital treatment. Zebularine, an orally bioavailable cytidine analog with demethylating activity that has been extensively studied in vitro, circumvents the need for an intermittent in-hospital dosing schedule and allows for convenient daily dosing. The ease of use and flexible dose scheduling of an orally bioavailable demethylating agent offers advantages over injectable agents; thus, zebularine was investigated in laboratory dogs and tumor-bearing dogs [98]. Based on extrapolation from in vitro data and data in rodents, laboratory dogs were initially treated with 8 mg/kg to determine plasma pharmacokinetics and then daily with 4 mg/kg to determine toxicity. Interestingly, and unexpectedly based on previously published in vitro and in vivo rodent data, daily treatment with 4 mg/kg zebularine resulted in severe neutropenia that was resolved with discontinuation of the drug, but required supportive care. Maximum plasma concentrations following treatment with 8 mg/kg and 4 mg/kg were 23 ± 4.8 *μ*M and 8.6 ± 1.4 *μ*M, respectively, which were much lower than concentrations previously reported in vitro and in vivo. Neutropenia was not observed in a cohort of three tumor-bearing dogs treated with 4 mg/kg once every 21 days, including two dogs with InvUC.

The study of zebularine in laboratory dogs and tumor-bearing dogs demonstrates the utility of studies in a canine model, as it was quickly established that zebularine concentrations previously achieved in vitro and in vivo in rodents could not be achieved safely in a veterinary clinical setting in dogs. However, two dogs with InvUC experienced stable disease for 12 and 18 weeks while receiving treatment with zebularine (4 mg/kg q21 days), suggesting a clinical benefit even at doses much lower than those previously reported in vitro and in vivo in rodents and primates [109]. A subsequent dose escalation trial in dogs with InvUC is in progress with the aim of defining a tolerable, long-term daily treatment schedule, with promising results to date. These studies demonstrate the promise for demethylating treatment strategies in InvUC as well as findings in dogs that would be expected to be translated into beneficial effects in humans and inform the design of human clinical trials.

## 5. Challenges to Be Met in Applying Naturally Occurring Canine Cancer Models

There is compelling evidence for the value of studies of pet dogs with naturally occurring cancer as a complementary animal model for improving the success rate in human trials, thus helping reduce the morbidity, mortality, and health care cost associated with cancer treatment, as well as greatly reducing the overall cost of the drug development process. There are, of course, challenges to be met in applying pet dog models. First, the characterization of canine cancer at the molecular level is in the early stages. While it is further along in some cancers, such as InvUC [19, 58, 71–73, 75, 110], than in others, there is still much work to be done. Second, the “canine model system” has not yet been fully tested to prove that the outcome in dog studies will predict the outcome in human studies. There is early evidence of this [88, 94], and more work is in progress, but, currently without this proof, major pharmaceutical companies have hesitated to invest in canine studies. And, without the funding, the proof of concept work in dogs cannot be done. An appropriately powered, randomized three-arm treatment trial in dogs, depending on the agent being tested and numbers of dogs needed, would cost a fraction of a similar human trial, but would cost much more than a laboratory rodent study. In addition, clinical trials in dogs can take a few months to two to three years, while the rodent studies are accomplished more quickly. Third, the number of veterinary clinician scientists and programs with the requisite skills, knowledge in comparative oncology, and specific tumor caseload would be required to grow should the demand for comparative oncology trials escalate. In addition to academic programs, there are other programs in place to facilitate canine clinical trials including the Comparative Oncology Trials Consortium directed by the Comparative Oncology Program at the National Cancer Institute [111]. Another consortium, the Canine Comparative Oncology Genomics Consortium (CCOGC) [112], sponsored by the National Cancer Institute, the American Kennel Club Canine Health Foundation, the Morris Animal Foundation, and Pfizer, is facilitating access to tumor and normal tissues and other samples from dogs through a biospecimen repository.

With the tremendous benefits to be gained from the full utilization of naturally occurring canine animal models of cancer, the challenges described above can certainly be met with a concerted effort in this area. As therapeutic drugs continue to become specifically targeted or engaged in complex interactions with the intact immune system, naturally occurring canine models that replicate key features of human cancer (cancer heterogeneity, molecular complexity, invasion, metastasis, and immune cell response) are uniquely positioned for the investigation of new therapeutic drugs and to complement work in traditional animal models. With the reports of immune checkpoints in canine tumors that are critical in human cancer, there is considerable interest in developing canine-specific therapeutic antibodies to block these checkpoints [62, 113–116]. The promise of comparative oncology research to improve the success of human clinical trials has never been more important.

## Figures and Tables

**Table 1:** Similarities and differences in naturally occurring invasive urothelial carcinoma between dogs and humans.

Similarities between dogs and humans
Physiological age of onset and clinical symptoms
Pathologically high grade, heterogenous cancer
Molecular subtypes (e.g., luminal, basal)
Epigenetic features
Shared molecular targets (e.g., EGFR, CDKN2B, PIK3CA, BRCA2, and NFkB)
Local cancer invasion into the bladder wall
Distant cancer metastases in ≥50% of subjects
Response to chemotherapy (e.g., cisplatin, carboplatin, and vinblastine)
Differences between dogs and humans
Sex differences (male : female ratio 2 : 1 in humans, 0.5 : 1 in dogs; although most dogs studied had been spayed or neutered)
Tumor location in bladder (more often trigonal in dogs; more variable in humans)
Dog tumors possess dog homologue of BRAF V600E mutation common in human melanoma (human InvUC has other variants in MAPK signaling)
